# Traditional knowledge and practice of the Triassic variegated clay from Silesia (Krasiejów), Poland, in human medicine

**DOI:** 10.1186/s13002-021-00437-0

**Published:** 2021-02-17

**Authors:** Izabela Spielvogel, Krzysztof Spałek, Krzysztof Badora, Jarosław Proćków

**Affiliations:** 1 Department of Physiotherapy, Institute of Physiotherapy, Opole University of Technology, Prószkowska 76, 45-758 Opole, Poland; 2 Institute of Biology, University of Opole, Oleska 22, 45-052 Opole, Poland; 3 Department of Economy, Institute of Socio-Economic Geography and Spatial Management University of Opole, Oleska 48, 45-052 Opole, Poland; 4 Institute of Environmental Biology, Faculty of Biology and Animal Science, Wrocław University of Environmental and Life Sciences, Kożuchowska 5b, 51-631 Wrocław, Poland

**Keywords:** Triassic variegated claystone, Peloids, Medicinal clays, Medical treatment, Ethnomedicine

## Abstract

**Background:**

Krasiejów clay (in German: Krascheow) became famous following the discovery of numerous fossilised bones of Upper Triassic amphibians and reptiles, which have been extracted from clay deposits since the 1980s. These organic remnants remained in Krasiejów clay due to the large amount of slime deposits and the optimal concentration of basal mineral salts.

The main aims of the paper are to determine the historical evolution of the use of clay in Silesia for therapeutic purposes and to provide a summary of the historical uses of Krasiejów clay as a medical treatment, based on the knowledge of the local population.

**Methods:**

The mode of utilisation of Triassic variegated claystone treatment from Krasiejów was surveyed based on oral communication with local people. This information was collected over the last 35 years (1982–2017) by interviewing those who used clay as a traditional remedy, especially the eldest residents. Each resident was interviewed several times regarding the healing properties of Krasiejów clay, including the causes and symptoms of the ailments treated, mode of clay preparation, application methods, and dosage through semistructured interviews.

**Results:**

Clay from Krasiejów was used in medicine after proper preparation. The clay underwent a complicated preparation process before the use in wraps, compresses, poultices, and baths as a skin peeling agent and even as a potion to be drunk. All recorded applications, diseases, and ailments to which it were applied are described here in detail, divided into treatments with warm, and cold clay.

**Conclusions:**

Krasiejów variegated claystone had different benefits depending on the form in which it was used; different diseases were treated with warm and cold clay. According to informants, many of these diseases have been successfully treated, in particular eczema of various origins, purulent ulcers on the skin, and following internal use, digestive ailments. According to informants, in cases where the disease could not be cured, for instance, psoriasis, a significant improvement in the condition of the skin was visible in a short period of time. Clay from Krasiejów should be subjected to more detailed physicochemical analyses to determine its exact chemical composition and healing properties.

**Supplementary Information:**

The online version contains supplementary material available at 10.1186/s13002-021-00437-0.

## Background

### Medicinal clay in ancient times

Due to its therapeutic properties, the clay has been used in the medicine worldwide since ancient times, which was even mentioned by Aristotle (384–322 B.C.) [1–3]. The therapeutic use of clay (“terra sigillata medicorum”) by potters on human and pet animals was known until the nineteenth century in places of different cultures or religions around the world [2, 4–11]. Bernard Palissy (1510–1589), a French potter and geologist, attempted to explain the action of “terra sigillata Lemnia” in his work entitled “Discours admirables” from 1580, concluding that this type of clay may also be present in other parts of the world, an assumption which was later proven correct [12]. He suggested that it may be present in Turkey; however, a similar clay was found in the sixteenth century in Central Europe, specifically in Silesia, being part of the Habsburg Empire at that time [1, 6, 9, 12–14].

### Medicinal clay in Silesia: “terra sigillata Silesiaca”

**Fig. 1:**
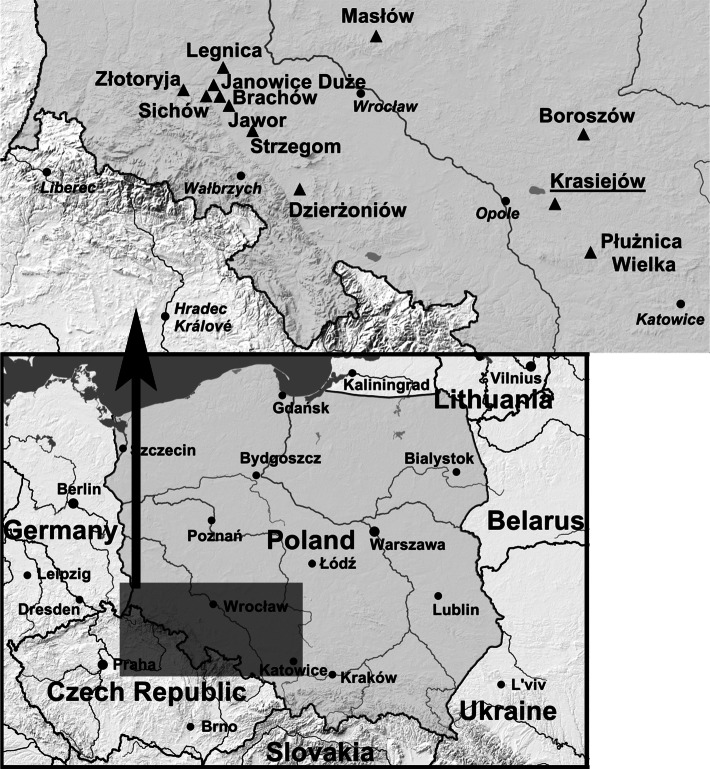
Localities (black triangles) of medicinal clays in Silesia, Poland. Silesian medicinal clay “terra sigillata Silesiaca”: clay from Strzegom (in German: Striegau) (“terra sigillata Strigoniensis” also called “Monits Acuti”) observed on three mountains within the Strzegom range: Góra Szeroka (Breitenberg), Góra Świętojerska or Góra Bazaltowa (Georgenberg), and Góra Krzyżowa (Kreuzberg); clay from Legnica (Liegnitz) (“terra sigillata Lignicensis”); clay from Złotoryja (Goldberg) (“terra sigillata Goltbergensis”). Additional localities of Silesian medicinal clay, according to the imperial order in 1685: Janowice Duże (Groß Jänowitz), Sichów (Seichau), Jawor (Jauer), Dzierżoniów (Reichenbach), Masłów (Massel), and Brachów (Brechelwitz) [currently it does not exist—flooded with water in the Słup retention tank]. Opole Silesia medicinal clay (name underlined): clay from Krasiejów. Upper Silesian medicinal clay: red clay from Płużnica Wielka (Groß Pluschnitz) (“terra sigillata Magni Plussnicensis”) and white clay from Boroszów (Boroschau) (“terra Nobarsovensis”). Part of the map marked with a gray rectangle is enlarged on the right

Caspar Schwenckfeld (1563–1609) was a spa doctor from Cieplice (in German: Bad Warmbrunn), Silesia, regarded as one of the outstanding Silesian scientists of the Renaissance, who also mentioned “terra sigillata Silesiaca” (Fig. 1) in his monograph “Stirpium & Fossilium Silesiae Catalogus” [15].

Silesia is a region with beneficial conditions for the presence of clay, including those with potential therapeutic efficacy, due to its very diverse and mosaic geological landscape [16]. There exist Precambric, Ordovician, Devonian, Carboniferous, Permian, Triassic, Cretaceous, Tertiary, Neogene, and Quaternary surface formations, with complexes of clay from weathering (kaolinic clays) or sea and limnic sedimentation. The oldest clays are from the early Mesozoic era, and the youngest from Pleistocene glaciation and river formations. Due to the great diversity of mineralogical and petrographic rocks in Silesia, there is a large diversity of clays resulting from their weathering. Rocks forming the Sudety Mountains (in German: Sudeten) and Przedgórze Sudeckie (in German: Sudetenvorland) foreland are also found in the south part of Silesia [17]. The majority of this region contains Paleozoic, magmatic, volcanic, and metamorphic rocks, the weathering of which has resulted in the present kaolinic clays. Weathering in the warm climates of the Mesozoic and early Cenozoic eras was beneficial to these clays, which are present, among others, in the Strzegom (in German: Striegau) and Złotoryja (in German: Goldberg) regions (Fig. 1), where their therapeutic use has been documented [6, 12, 15, 18]. The northern part of Silesia was formed by the deposits of Fore-Sudetic Monocline, around which clays, silts, claystones, and mudstones of the Triassic (upper Triassic mainly) and Jura (lower Jura mainly) eras are found, which were deposited in the sea and limnic environments, in addition to being formed from decayed limestone (basal kaolinic clay). Triassic and Jura clays were used in therapeutics from at least the seventeenth century and were known in Europe as the layers present within the Olesno and Płużnica Wielka regions (Fig. 1) [1, 12, 13, 18].

The first use of clay deposits in medicine in Silesia, “terra sigillata Silesiaca”, has been dated to 1550 AD, and the oldest written information related to this use has been dated to 1586 AD [1, 6, 9, 12, 13, 18]. These deposits were described by their discoverer, the Renaissance physician and geologist in Jelenia Góra, Johann Schulz (1531–1604), also known as Johannes Scultetus Trimontanus or Johannes Montanus, in “Judieum de terra sigillata strigoviensis” published in Nuremberg [1, 6, 9, 12, 13, 18]. His work described three types of Silesian medicinal clay “terra sigillata Silesiaca”: clay from Strzegom “terra sigillata Strigoniensis” also called “Monits Acuti”, clay from Legnica (in German: Liegnitz) “terra sigillata Lignicensis”, and clay from Złotoryja “terra sigillata Goltbergensis” (Fig. 1). Thanks to his discovery, Montanus became a famous and the personal doctor of Emperor Rudolph II [11, 13, 18].

Medicinal clay is formed by the accumulation of a mixture of minerals such as smectite, bentonite, montmorillonite, kaolinite, illite, and metahaloisite, with impurities of other minerals and fractions, resulting from the chemical weathering of rocks and the sedimentation of detritus. The quantitative ratios of individual minerals are very diverse, similar to the diverse chemical composition, and mainly depend on the type of rock from which the clay was weathered, the sedimentation conditions, and the processes that occur after sedimentation. Medicinal clays derived from basalt weathering have different properties to those derived from the weathering of granite, amphibolite, gneisse, or limestone. In Johannes Schulz’s description of “terra sigillata Silesiaca,” he distinguished two types of clays: red and white. He defined these clays as gold that were changed to red clay due to the sun, “Axungia Solis”, and white clay due to the moon, “Axungia Lunae” [6, 9]. Montanus’s descriptions focused mainly on therapeutic indications for the use of Strzegom clay. He observed its occurrence on three mountains within the Strzegom range: Breitenberg (Góra Szeroka), Georgenberg (Góra Świętojerska or Góra Bazaltowa), and Kreuzberg (Góra Krzyżowa) [6, 18]. In 1580, Silesian clay was already known as a therapeutic agent for canine rabies in the Land of Hessen in West Germany [12], and by 1852, it could be purchased at markets in Frankfurt. In 1618, “terra Silesiaca” was first named in the “Pharmacopoeia Londinensis” alongside the classic clays of the Mediterranean region [1, 6]. In 1589, the Strzegom municipality obtained the imperial privilege to extract clay from these three deposits, and seals with a representation of the three Strzegom hills were used to confirm its authenticity. In 1685, the imperial order was introduced for the absolute sealing of clay obtained from other Silesian deposits located in Strzegom (in German: Striegau), Złotoryja (in German: Goldberg), Janowice Duże adjacent to Legnica (in German: Groß Jänowitz), Legnica (in German: Liegnitz), Sichów (in German: Seichau), Jawor (in German: Jauer), Dzierżoniów (in German: Reichenbach), Brachów (in German: Brechelwitz), and Masłów adjacent to Trzebnica (in German: Massel) (Fig. 1) [1, 12]. The following clays were known in Upper Silesia: red clay from Płużnica Wielka (in German: Groß Pluschnitz) and white clay from the Olesno region (in German: Rosenberg), i.e., from Boroszów (in German: Boroschau), called “terra Nobarsovensis.” This clay was discovered in 1700 by Gottfried Wahl who came from Świebodzice (in German: Freiburg) and was a councillor in Oleśnica (in German: Oels). In 1714, Paul Jakob Marperger mentioned its antitoxic and antipyretic action, and it was also indicated for stomach disorders [12, 18]. From the sixteenth to eighteenth centuries, dishes were made from “terra sigillata Silesiaca.” In the Masłów region, during the eighteenth century, pills were made from the clay and used as a medicine, which was named Töppelberg, after the place where it was discovered; the hill of Töppelberg, located in the west. In the eighteenth century, Silesian clay was still used as a medicine, and according to Brunner, its health benefits had been known since prehistoric times [1, 12]. According to Marpergra’s description, Strzegom clay was used for slow-healing wounds caused by bites. It was used externally in the form of compresses and poultices, as well as internally in the form of a liquid solution with water, wine, beer, and vinegar, or in the form of pills. A compress could also be made after mixing the clay with the saliva of an ill, fasted patient who had not eaten onions, garlic, or pork for a few days [1, 12]. It was indicated for the treatment of wounds after being bitten by a dog infected with rabies. Other indications include intestinal disorders, bloating, constipation, eye disease, nosebleeds, wound bleeding, headaches, fever, and cholera [1, 9, 12]. Indications for the therapeutic use of the clay from the Silesian deposits were also described in “Rariora naturae & artis, item in re medica; oder, Seltenheiten der Natur und Kunst des kundmannischen Naturalien-Cabinets, wie auch in der Artzeney-Wissenschafft” by a physician of the Hochberg family, Johann Kundmann [13]. According to Kundmann, dishes made from “terra sigillata” clay extracted from the Silesian deposits had therapeutic properties in folk medicine; it was believed that anyone who drank from a clay dish every day would avoid food poisoning.

According to Kundmann’s description, in 1633, the red clay from Płużnica Wielka adjacent to Strzelce Opolskie (in German: Gross Strechlitz), “terra sigillata Magni Plussnicensis” (Fig. 1), helped the local community as a preventive agent to control a cholera epidemic and mass cattle deaths in Upper Silesia [1, 12, 13]. Andreas von Wehner, an aristocrat and councillor from Wrocław (in German: Breslau), was granted permission to extract therapeutic clay from Płużnica Wielka, which he sealed with a crescent and three arrows. In addition to external compresses, the clay was also mixed with wine or beer by the local population and consumed orally. For use in animals, the clay was mixed with vinegar and ash [13]. In Strzegom, the production of the drug was discontinued during the second half of the eighteenth century. Today, collections of clay pills originating from Strzegom and other regions of Lower Silesia are placed, among others, in the Museum in Wałbrzych (in German: Waldenburg), the Museum of Old Trading in Świdnica (in German: Schweidnitz), the Mineralogical Museum of the University of Wrocław, and the Museum of Pharmacy in Heidelberg.

### Origin of medicinal clays

Based on the research regarding medicinal clays and the scale of source materials, including marked seals on clay deposits, the following best known clays used in medicine can be determined: clay from Lemnos (“terra Lemnia,” “terra sigillata”), clay from Malta (“terra Malitea”), clay from Palestine (“terra Hierosolymitanae”), clay from Armenia (“terra Armenica”), clay from Turkey (“terra Turcica”), clay from Silesia (“terra Silesiaca”), clay from Livonia (“terra Livonica”), Italian clay (“terra Florentina”), Portuguese clay (“terra Portugallica”), Spanish clay (“terra Hispanica”), and clays from Kisameet Bay in Canada, Côte d’Ivoire, Scandinavia, and Persia [2, 6, 11, 19, 20].

### Mechanism of action and scientific evidence of medicinal clays used in traditional therapy

Although clays were used for medicinal purposes for millennia, they remained largely unexamined in terms of their mechanism of action and potential benefits in medicine. In traditional human medicine, the clay has been used both externally and internally, for instance, as an aseptic, astringent, and absorbing agent. Recently, however, there has been an increased interest in the geochemical properties of these minerals with respect to antibacterial and anti-inflammatory actions [2, 8, 19–23]. The emergence of antibiotic-resistant bacteria has accelerated the search for new sources of antibacterial compounds. Bacteria quickly gain resistance to antibiotics that target specific cellular mechanisms, DNA replication, and the synthesis of proteins and cell walls. As a result, scientists have become interested in age-old traditional mineral-based therapies against bacterial infections. The Kisameet clay solution used for centuries by the Heiltsuk Indian tribe (now in British Columbia, Canada) has proven its effectiveness against 16 strains of multidrug-resistant nosocomial infection-causing bacteria including *Enterococcus faecium*, *Staphylococcus aureus*, *Klebsiella pneumoniae*, *Acinetobacter baumannii*, *Pseudomonas aeruginosa*, and *Enterobacter* sp. [19, 20]. The results suggest that clay may be used as a therapeutic option to treat serious infections caused by these bacteria.

Studies by Williams and Haydel [2] suggest that clay minerals used by Afro-Americans to treat *Buruli* ulcers, known as “flesh eating bacteria,” speed up the process of wound healing in persons infected with bacillus *Mycobacterium ulcerans**.* Morrison et al. [21] proved that certain clays kill antibiotic-resistant bacteria, including methicillin-resistant *Staphylococcus aureus.* Oregon blue clay has shown strong antibacterial action, containing soluble reduced metals and clay minerals that absorb cations, which ensure prolonged metal release and the production of toxic hydroxyl radicals. Efimenko et al. [24] described the anti-inflammatory action of clay from Tambukan lake in Russia. For clays used to treat chronic slow-healing wounds, pH and Eh (redox potential) buffering mechanisms seem to be crucial for the curative capacity as an alternative to antibiotics in medicine [19–21, 24, 25].

Moreover, detoxification as an adaptive function of geophagy is demonstrated from field and historical data associating clay consumption [26]. These results were confirmed by reports of geophagy in nonhuman primates. Thus, the inorganic component of the chemical environment deserves increased attention from chemical ecologists [26]. Other hypotheses have been advanced to explain geophagic behavior, the main ones being alleviation of gastrointestinal upsets such as diarrhoea, supplementation of mineral nutrients, and as a means of dealing with excess acidity in the digestive tract [27]. Geophagy has its human origins in tropical Africa from where the practice migrated to become an almost universal phenomenon. Today, geophagy has the widest distribution amongst the world’s poorer or more tribally orientated people [28].

### Aim of the study

This paper presents a survey to determine (1) the historical evolution of the use of clay in Silesia for therapeutic purposes and (2) the use of Krasiejów clay and an analysis of its therapeutic patterns. We provide a mode of utilization and indications for the therapeutic use of Krasiejów clay in folk medicine based on oral communication with local residents. In particular, the present study may guide research on novel mineral therapeutic agents, inform safety evaluations, help to prove the traditional use in the context of drug regulation [29, 30], and determine the historical evolution of clay use for therapeutic purposes in Silesia. To date, clay from Krasiejów has not been described in the literature. Thus, the purpose of the data collection, done over 35 years, is to show the medical properties of Krasiejów clay. This paper addresses the following research questions: (1) According to the informants and patients, what types of diseases were cured with Krasiejów clay? (2) According to them, does it seem to be worth investigating the healing properties of these clays in the future?

## Materials and methods

### Study area

**Fig. 2:**
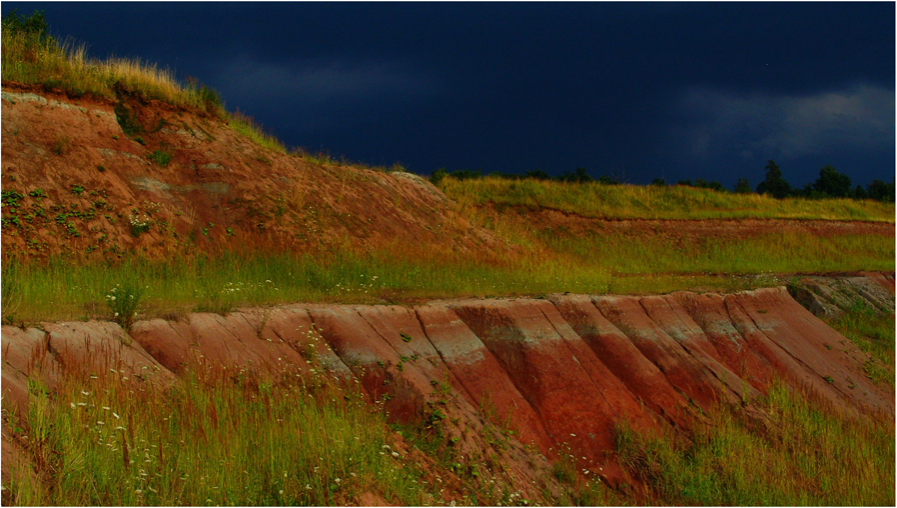
Deposits of medicinal clay in the mine in Krasiejów (1990s, photo by Krzysztof Spałek)

Krasiejów became famous for the discovery of numerous fossilized bones of Upper Triassic amphibians and reptiles, which have been extracted from clay deposits in the closed mine since the 1980s (e.g., [31, 32]), including no less than 14 taxa new to science [33]. In the Krasiejów area, approximately 225 million years ago, there were swampy areas located on the shore of a large lake, reaching Olsztyn in northern Poland and western France [34]. A wide river delta also entered the lake in this area. In Krasiejów, there are layers of variegated cherry-red silt and claystones that are deposits of late Triassic rivers and lakes. These deposits sedimented under diverse conditions, with periods of marine transgression and terrestrial episodes, and anaerobic reduction conditions in mud swamp marginal lakes were widespread. The existence of Triassic clays was discovered by the German geologist and paleontologist, Carl Ferdinand von Roemer [35], at the bottom of Mała Panew (in German: Malapane) river near the steel plant in Ozimek (in German: Ozimek). Numerous organic remnants remained in Krasiejów clay due to the optimal concentration of basal mineral salts and a large amount of slime deposits. A Triassic river played an essential role in this process. In the deposits at the bottom of lakes or rivers, bacteria decompose organic substances deposited on silt or sand, using oxygen and emitting poisonous hydrogen sulphide. On the site of the former clay mine in Krasiejów, there are several types of coloured silt forming layers of different thicknesses (Fig. 2), which contain relatively large amounts of aluminium oxide (13.82%), calcium (6.90%), iron (5.72%), magnesium (3.50%), potassium (2.77%), and silicon (2.57%) [36]; therefore, after proper preparation, these clays can be used as medicinal treatments. It was also confirmed that most families in Krasiejów had the clay in the home medicine cabinet from roughly 1900 to 1960, and the peak of its use came after the opening of the brickyard.

### History of the region in a brief

The village of Krasiejów (until 1933 Krascheow, from 1933 to 1945 Schönhorst) is situated in Silesia, more precisely in Upper Silesia, which until the mid-sixteenth century was under the rule of the Kingdom of Bohemia. After 1526, Silesia came under the rule of the Habsburg Monarchy, and then, as a result of the Silesian Wars in 1740–1742, it became part of the Kingdom of Prussia. After World War II, pursuant to the provisions of the Yalta and Potsdam Conferences (both in 1945), Silesia was incorporated into Poland. The German population inhabiting these areas so far was displaced to West Germany (about 3 million people), and these areas were settled by Poles from the so-called Eastern Borderlands (areas of today’s Ukraine). The planned displacement of the Germans from Silesia began in the fall of 1945, and the climax was in the years 1946–1947 [37, 38]. The displacements, however, were related to certain restrictions: they did not include, for example, professionals whose knowledge was necessary to maintain the continuity of the operation of factories or municipal facilities (mines, coking plants, waterworks, power plants) and the population of the Polish-German border using the Silesian dialect. This group of people lived mainly in the border and industrial area of Upper Silesia (the area east of the Nysa Kłodzka river). Therefore, a significant part of the inhabitants of Upper Silesia was not displaced, and after the war, they had two citizenships—Polish and German. The population of Krasiejów is 90% indigenous, living here for generations—that is, the village is not actually inhabited by immigrants arrived after 1945. There is a German minority in the village, and bilingual (Polish-German) place names have been introduced in the region [37, 38]. The unique knowledge of folk healing practices with the use of local variegated clay presented in this paper has thus survived continuously, thanks to the oral tradition passed down from generation to generation. Currently, the method of clay treatment by the inhabitants is not used, and knowledge of these practices is in danger of being forgotten as it has not been previously written down.

### Methods: sampling and interview

Since it has been confirmed that Krasiejów clays have medicinal properties, we surveyed the mode of utilisation of Triassic variegated claystone treatments from Krasiejów based on oral communication with local residents. This information was passed down through the generations starting from the nineteenth century. The most knowledgeable person regarding these matters was Mrs. Bühl from Szczedrzyk (in German: Sczedrzik) (9 km NW of Krasiejów), a local herbalist who died at the beginning of the twentieth century (a tombstone still existed in 1970 at the local cemetery in Krasiejów; however, it did not survive to date). The grandmother (no current inhabitants of Krasiejów could remember her name) and mother (Marie Gawlik) of the later owner of the brickyard (Johann Gawlik) directly benefited from Mrs. Bühl’s knowledge. Johann Gawlik was particularly interested in the properties of clay, especially since at that time there were no doctors or pharmacies in the villages. Johann Gawlik was the owner of the Krasiejów’s tavern, wedding hall, several shops, and later (from the 1930s) the brickyard and was therefore an important person in the local community and well respected in Krasiejów. Most of the residents of Krasiejów and neighboring villages drew their knowledge regarding medicinal clays directly from the Gawlik family, including Paul Kapica. He lived in the nearby village of Spórok (in German: Sporok) (5 km SE of Krasiejów), and the residents of Krasiejów were also treated by him.

The knowledge presented in the present paper was collected by Krzysztof Spałek, who came from Krasiejów, during the past 35 years (1982–2017). (Krzysztof Spałek became interested in these issues after he witnessed the treatment of his father’s psoriasis with hot clay wraps.) He interviewed the indigenous inhabitants of Krasiejów and the surrounding area who lived at that time, especially the eldest people, including the family of the former owner of the brickyard, its employees, and residents of the village who used clay as a traditional remedy. All possible people have been asked to provide the relevant information (53 informants were interviewed only, but they were all people who could be questioned on this matter). As Krzysztof Spałek (who interviewed the informants) passed away in the meantime, we were not able to determine the gender structure of respondents. However, statistical data from the surveyed villages and surrounding area were at the level of approximately 51% women and 49% men. The sex of the informants was not given preference, and all of them were interviewed (therefore, it is very likely that ca. 27 women and 26 men were interrogated). The age group of the informants varied between 60 and 81 years of age, although most of them were between 70 and 81 years of age. This age structure cannot be confirmed in more detail, because we no longer have access to the surveys made by K. Spałek.

The informants were questioned in their local language (Polish Silesian) about the healing properties and the use of Krasiejów clay, including the causes and symptoms of the ailments treated, mode of clay preparation, application methods, and dosage. The information was collected from the informants through semistructured interviews (using a guided questionnaire—see it in Additional file 1 as it was additionally translated from Polish into English for this paper). The questionnaire contains information on the form of clay that was used for medicinal purposes, admixtures added to the clay, and kinds of treatments used with the clay, as well as questions on the form of warm and cold treatments and diseases treated these ways, the process of clay preparation (including purification processes), how were the informants familiar with the treatment of clay from Krasiejów, and for how long in their families was clay used for healing purposes and whether the treatments were effective.

All recorded information obtained from the local community of the Krasiejów area was compiled and used in the preparation of this article. Before interviewing any person, the objectives of the study, method and planned use of the information were explained to them, and permission to conduct the interview was sought. Oral informed consent to share their knowledge about the clay they used to cure the disease was obtained from each participant prior to conducting the interview. The willingness to participate in the research was crucial. Each resident was interviewed several times during the study period to validate the information provided. Following Alexiades and Sheldon [39], responses that contradict each other should not be considered for analysis (i.e., only those formulations having consistency can be considered); however, throughout the entire research period there were only three cases of contradicting responses (one, two, and two responses in three different questionnaires); thus, only the responses having consistency were used. The low value of contradicting responses is possible, especially if the research group is not large (53 informants).

The data obtained were especially valuable, since the residents who had the most knowledge regarding these treatments were no longer alive. Thus, we provide recipes that were used directly by local residents based on oral communication. The study is then based mostly on interview data, as we did not observe actual therapeutic practices/treatments involving clay because they are not normally used at this time. Namely, an increasing number of doctors and pharmacies in the villages contributed to the disappearance of the use of Krasiejów clay for medicinal purposes. There are also no written prescriptions on the ancient use of clay from Krasiejów, so it cannot be traced in more detail. This is the only data on this topic.

In addition, an exhaustive review of old prints on ethnopharmacology in the context of Silesian clay use was carried out. All possible literature items have been used, including German, Polish, and English works.

## Results

Krasiejów clay was used by the local population as compresses consisting of powdered clay or ground crusts of burnt dishes diluted with water, denatured alcohol, rectified spirit, vinegar, sour milk, chicken egg white, and sometimes the addition of a spider’s web. Clay that underwent a complicated preparation process was most commonly used. Clay intended for treatment was supplied in the autumn and placed in the yard to mature during the winter. In the spring, when it was adequately warmed by the sun, its proper preparation began. It was crushed, comminuted with a hammer, and subsequently manually kneaded for roughly 2 h until the plasticity was sufficient. It has been used externally for wraps, compresses, poultices, and baths as a skin peeling agent. The worked clay was generally used to treat burns, fractures, and dislocations (with the addition of vinegar), swelling and edema, including sore gums and toothache (with the addition of vinegar, spirit, and water in proportions of half and half as a wrap), fever, ulcers, boils, abscesses, slow-healing wounds, neuralgia, bruises, insect bites, animal bites, lichens, and eczema. Baths in clay diluted with water, were also taken, and the water-clay solution, standing one day, was drunk for the treatment of stomach pains. These were the most common diseases and ailments treated with this clay in Krasiejów.

### Forms of preparation and administration of Triassic variegated claystone treatment from Krasiejów

Procedures used for the treatment with clay in Krasiejów were carried out in accordance with the following mode:

### Full bath, ¾ bath, and ½ bath

Baths were taken in a highly diluted clay solution at a temperature of approximately 37 °C for 20 min (warm treatment) or at approximately 32 °C (cold treatment) for 5 to 7 min.

### Partial bath of the upper or lower limbs

Baths were taken at a temperature of 37–38 °C for approximately 10 min (warm treatment) or at 10–15 °C (cold treatment) for 5 min.

### Full wrapping

Depending on the indication, the wrap was made from warmed or cooled clay paste. A wool blanket with foil and linen were placed on a table, and the body was covered with 1 to 2-cm thick clay. The body was subsequently wrapped in a foil, linen fabric, and blanket. During hot wraps, a cold compress on the forehead or around the heart was indicated. The time of treatment at a temperature of approximately 37 °C was 20 to 30 min (warm treatment) or at a temperature of 10–15 °C was approximately 10 to 15 min (cold treatment).

### Partial wrapping

A partial wrap was performed in the same way as a full wrap, but was only placed on a particular area such as the cervical spine or upper limb. The durations of warm and cold treatments and clay temperature were the same as shown above.

### Pressing warm clay paste

Treatment was based on pressing a clay paste at a temperature of approximately 45 °C for 10 to 15 min and was used in rheumatic diseases and arthroses in the interphalangeal joints of the upper limb.

### Compresses and poultices

These were made using a clay plaster of 20 × 30-cm and 1 to 2-cm thick, warmed to a temperature of 45 °C or cooled to 10 to 15 °C, and applied to the selected area of the body. Following treatment, the paste was washed out. On average, the series included 10 to 20 treatments.

### Discussion and summary of the indications of clay use

Clays, due to their healing properties, have been used in the medicine in all parts of the world since the earliest times. For example, the Kisameet clay slime, used for centuries by the Heiltsuk Indian tribe, has proven effective against sixteen strains of multidrug-resistant bacteria that cause most nosocomial infections [19, 20]. A study by Williams and Haydel [2] shows that the clay minerals used by Afro-Americans to treat a Burula ulcer known as “flesh-eating bacteria” promote wound healing. In other studies, Morrison et al. [21] confirmed that Oregon Blue Clay showed a strong bactericidal effect—it kills, for example, methicillin-resistant golden staphylococcus. The clay from Krasiejów seems to be similarly useful in various therapies, as summarized below.

### Indications for treatment with warm clay

Heated variegated mudstones from Krasiejów were traditionally used in the following diseases: skin diseases including psoriasis, conditions after injury and strains of the locomotor system, rheumatic diseases, disorders of the arterial, venous, and lymphatic circulation in the limbs, neuralgia and arthroses, rheumatoid arthritis in remission, and ankylosing spondylitis in remission.

### Indications for treatment with cold clay

Cold variegated mudstones from Krasiejów were traditionally used in inflammatory states of the skin, veins and lymphatic vessels, rheumatic diseases of the joints, dislocations, sprains, injuries, crushes, hematomas and burns, infectious diseases with fever, insect bites, animal bites, and slow-healing, suppurative wounds, and ulcers.

As 94.34% analyzed questionnaires were consistent, this study extends existing scientific knowledge on the therapeutic use of clays, as Krasiejów clay was used with additional disease units before it was known (compare e.g., [2, 5, 7, 8, 10, 11, 14, 19, 20, 24]). Namely, to date, the clays were used mainly as antibacterial agents, especially in the case of antibiotic resistance. They also help with arthritis and are generally used in pelotherapy, geotherapy, and paramuds. More generally, they are administered orally (as gastrointestinal protectors, laxatives, antidiarrhoeics) or as topical applications (dermatological protectors and cosmetics). Moreover, the applications of clay minerals in aesthetic medicine (to clean and moisturise the skin and to combat compact lipodystrophies, acne, and cellulite) are also known [14, 10].

Based on the unique information collected in this study, it is necessary to evaluate the antibacterial potential of clays from Krasiejów—their biological and enzymatic activity, because it gives an opportunity to learn about the real effect of clayey and their therapeutic activity. The obtained data can also be used to search for new drugs.

## Conclusions

Krasiejów variegated claystone has different benefits depending on the form in which it is used. Different diseases were treated using warm or cold clays. Many diseases were successfully treated, especially eczema of various origins, purulent skin ulcers, and after internal use, digestive ailments. In cases where the disease, e.g., psoriasis, could not be cured, a significant improvement in the skin condition was visible in a short period of time.

Unfortunately, the knowledge regarding Krasiejów clay is rapidly disappearing. The data obtained are especially valuable since the residents with the most knowledge regarding these clays are no longer alive (except for one person still living). Thus, here, we provide recipes that were used directly by local residents based on oral communication.

At present, clay from Krasiejów should be subjected to more detailed physicochemical analyses to determine its exact chemical composition and healing properties (and this should be the next step of future research).

## Supplementary Information

**Additional file 1.**

## Data Availability

All data are presented in this article.
