# Supplementary material for: Traditional knowledge and practice of the Triassic variegated clay from Silesia (Krasiejów), Poland, in human medicine
Source: J Ethnobiol Ethnomed. 2021 Feb 17;17:10. doi: 10.1186/s13002-021-00437-0 (PMC7890874; doi:10.1186/s13002-021-00437-0)
Supplement: Supplementary file 1 — Additional file 1. [file 13002_2021_437_MOESM1_ESM.doc]

**Ankieta – wykorzystanie gliny z Krasiejowa**

[Questionnaire – use of Krasiejów clay]

**1. W jakiej formie glina była używana do celów leczniczych ?**

[In which form was the clay used for medicinal purposes?]

**2. Czy do gliny były stosowane domieszki ? Jeśli tak to jakie ?**

[Have admixtures been added to the clay? If so, which kinds?]

**3. Jakie rodzaje zabiegów stosowano z użyciem gliny?**

[What kinds of treatments were the clay used for?]

**4. Czy glina stosowana była w formie zabiegów ciepłych?**

[Was the clay used in the form of warm treatments?]

a. tak / yes

b. nie / no

c. nie wiem / I do not know

Jeśli tak proszę przejść do pytania 4a. [If yes, please go to question 4a.]

**4a. Jakie schorzenia leczono z użyciem gliny z Krasiejowa w formie zabiegów ciepłych?**

[Which diseases were treated with clay from Krasiejów in the form of warm treatments?]

**5. Czy glina stosowana była w formie zabiegów zimnych?**

[Was the clay used in the form of cold treatments?]

a. tak / yes

b. nie / no

c. nie wiem / I do not know

Jeśli tak proszę przejść do pytań 5a i 5b. [If yes, please go to questions 5a and 5b.]

**5a. Jakie schorzenia leczono z użyciem gliny z Krasiejowa w formie zabiegów zimnych?**

[Which diseases were treated with clay from Krasiejów in the form of cold treatments?]

**5b. Proszę opisać proces przygotowania gliny.**

[Please describe the process of clay preparation.]

**6. Czy glina użyta do celów leczniczych przeszła proces oczyszczania?**

[Has the clay used for medicinal purposes undergone a purification process?]

a. tak / yes

b. nie / no

c. nie wiem / I do not know

**7. Skąd znane są Pani/Panu sposoby leczenia gliną z Krasiejowa?**

[How were you familiar with the treatment of clay from Krasiejów?]

**8. Od jak dawna w Pani/Pana rodzinie stosowana była glina z Krasiejowa do celów leczniczych?**

[For how long, in your family, has clay from Krasiejów been used for healing purposes?]

**9. Czy zabiegi z użyciem gliny z Krasiejowa były skuteczne?**

[Were the treatments using clay from Krasiejów effective?]

a. tak / yes

b. nie / no

c. nie wiem / I do not know

**--------------------------------**

10. Imię i nazwisko informatora ………………………………….

[Name and surname of the informant]

11. Wiek / Age ................

12. Płeć / Gender ..............

13. Wykształcenie / Level of education .............................................

14. Adres / Adress ………………………………………………………………………..

15. Zgoda na wykorzystanie danych (podpis ankietowanego) …………………………..

[Consent to the use of data (signature of the informant)]
